# Echocardiographic Changes in Chronic Kidney Disease Patients Before and After Arteriovenous Fistula Creation: A Prospective Study of Cardiac Remodeling

**DOI:** 10.7759/cureus.89693

**Published:** 2025-08-09

**Authors:** Muhammad Zaryab Haider, Ranjith Mule, Tabassum, Muhammad Anees Ur Rehman, Qurat Ul Ain, Muhammad Irfan Jamil, Tayyaba Arooj Mufti, Muhammad Ahsan Butt, FNU Anam, FNU Shahzeen

**Affiliations:** 1 Cardiology, Rawalpindi Institute of Cardiology, Rawalpindi, PAK; 2 Acute Internal Medicine, Blackpool Teaching Hospitals NHS Foundation Trust, Blackpool, GBR; 3 Emergency Medicine, Blackpool Teaching Hospitals NHS Foundation Trust, Blackpool, GBR; 4 Acute Medicine, Blackpool Victoria Hospital, Blackpool, GBR; 5 Acute Internal Medicine, Quaid-E-Azam Medical College, Bahawalpur, PAK; 6 Medicine, M. Islam Medical and Dental College, Gujranwala, PAK; 7 Medicine/Cardiology, University of Health Sciences Lahore, Lahore, PAK; 8 Nephrology, Lahore General Hospital, Lahore, PAK; 9 Cardiology/Medicine, Akhtar Saeed Medical and Dental College, Lahore, PAK; 10 Medicine, South Tyneside and Sunderland NHS Foundation Trust, Sunderland, GBR; 11 Medicine, Sayed Abdullah Shah Institute of Medical Sciences, Sehwan, PAK; 12 Internal Medicine, Jinnah Sindh Medical University, Karachi, PAK

**Keywords:** arteriovenous (av) fistula, cardiac echo, cardiac remodelling, chronic kidney disease (ckd), lvef (left ventricular ejection fraction)

## Abstract

Aim and background: Arteriovenous fistula (AVF) creation remains the preferred vascular access for hemodialysis in patients with advanced chronic kidney disease (CKD). However, the hemodynamic burden imposed by an AVF may lead to progressive cardiac remodeling, with implications for long-term cardiovascular morbidity. This study aimed to evaluate structural and functional cardiac changes following AVF creation in stage 4 and 5 CKD patients over a six-month follow-up using serial echocardiography.

Methods: This prospective study initially included 150 patients; however, 105 patients with complete data were analyzed at the six-month follow-up. Transthoracic echocardiography was performed at three time points: before AVF creation, at three months, and six months post-creation.

Results: At three months, transient improvements were noted in the left ventricular ejection fraction (53.16 ± 5.21% to 56.23 ± 4.85%, p<0.001), global longitudinal strain (GLS) (−15.21 ± 0.91% to −15.38 ± 0.89%, p<0.001), and diastolic indices. However, by six months, progressive cardiac remodeling was evident with significant increases in the left ventricular mass index (+20.63 ± 6.04 g/m², p<0.001), left ventricular end-diastolic diameter and left ventricular end-systolic diameter, and left atrial volume index (+7.50 ± 1.23 mL/m², p<0.001). Diastolic function deteriorated (early-to-atrial filling velocity ratio (E/A) and early diastolic to mitral annular velocity ratio (E/e′), p<0.001), pulmonary artery systolic pressure increased by +9.90 ± 1.42 mmHg, tricuspid annular plane systolic excursion declined (−2.07 ± 0.44 mm), and tricuspid regurgitation velocity rose (+0.38 ± 0.14 m/s). GLS showed a subclinical decline (−0.38 ± 0.11%, p<0.001).

Conclusion: AVF creation results in a biphasic cardiac response with early adaptive changes followed by significant structural and functional deterioration by six months.

## Introduction

Chronic kidney disease (CKD) affects an estimated 15-20% of adults worldwide, representing a major public health burden with considerable regional variation [1]. Cardiovascular disease is the leading cause of morbidity and mortality in CKD, exceeding the risk of progression to end-stage kidney disease. Epidemiological data indicate that up to 70-80% of patients with advanced CKD develop left ventricular hypertrophy, while diastolic dysfunction occurs in 28-70% and systolic dysfunction in 20-44% [2,3]. These cardiac changes increase in frequency and severity as kidney function declines, driven by both traditional risk factors and kidney disease-specific mechanisms, including chronic inflammation, volume overload, and mineral bone disorders [4].

Cardiac remodeling in CKD arises from a combination of hemodynamic, neurohormonal, and metabolic factors. Sustained hypertension and chronic volume overload promote left ventricular hypertrophy and chamber dilation. Anemia further amplifies cardiac output demands, while activation of the renin-angiotensin-aldosterone system and sympathetic pathways accelerates myocardial fibrosis. Inflammatory mediators and uremic toxins contribute to microvascular rarefaction and structural disarray [5-8]. Arteriovenous fistula (AVF), though the vascular access of choice for hemodialysis due to its superior patency and safe infection profile over catheter and grafts, introduces significant hemodynamic stress. The deviation of arterial blood directly into the venous system increases preload and cardiac output, increasing strain on the left ventricle. Early post-AVF changes typically include rises in left ventricular mass and left atrial dimensions [9,10]. These changes, particularly with high-flow or proximal fistulas, may worsen over time and predispose to progressive cardiac dysfunction, necessitating the importance of longitudinal cardiac monitoring in this population.

There is limited evidence describing the precise timeline of cardiac changes after AVF creation in advanced CKD patients not yet on dialysis, as most previous studies included individuals who had already started or transitioned to dialysis. This study specifically included stage 4 and 5 CKD patients prior to maintenance hemodialysis, allowing clearer evaluation of the direct effects of AVF on cardiac structure and function. In addition, less commonly used markers like global longitudinal strain (GLS) and detailed diastolic indices were systematically assessed, offering a more complete understanding of cardiac remodeling in this context [9-11].

Understanding the echocardiographic changes that occur following AVF creation is important for optimizing patient care in order to prevent cardiovascular complications. This study aimed to prospectively evaluate changes in cardiac structure and function, as assessed by echocardiography, before and after AVF creation. The objective is to identify the extent and pattern of cardiac remodeling.

## Materials and methods

A prospective cohort study was conducted at Lahore General Hospital, Lahore over a period of one year from August 2023 to July 2024, after taking approval from the institutional review board (IRB number: 156/10/2023). Informed consent was obtained from participants. One hundred and fifty adult patients aged ≥ 18 years with estimated glomerular filtration rate (eGFR) <30 mL/min/1.73 m², scheduled for AVF creation, were included. Exclusion criteria comprised pre-existing advanced heart failure (New York Heart Association class III-IV) or left ventricular ejection fraction (LVEF) less than 35%, history of previous AVF or graft, severe valvular heart disease, congenital cardiac anomalies, recent myocardial infarction or cardiac procedures within the preceding three months, and inadequate echocardiographic window.

Patients were screened during routine clinic visits, and those scheduled for AVF creation were invited to participate. Baseline data were collected within one week before AVF surgery, including age, sex, CKD stage, eGFR, duration of kidney disease, blood pressure, and history of diabetes, hypertension, and ischemic heart disease.

Transthoracic echocardiography was performed by an experienced cardiologist within seven days before AVF creation and repeated three months later, using a Philips EPIQ 7C ultrasound system (Philips Ultrasound Inc., United States) with a 2.5-3.5 MHz phased-array transducer in standard two-dimensional (2D), M-mode, and Doppler imaging modes, with the same machine and operator maintained throughout to ensure consistency and minimize inter-observer variability. Echocardiographic parameters were assessed in accordance with the American Society of Echocardiography guidelines. The specific variables measured, along with their reference ranges and units, are summarized in Table 1. All echocardiographic assessments were performed by the same experienced cardiologist using the same ultrasound machine at each scheduled time point to ensure consistency and minimize measurement variability [12].

**Table 1:** Echocardiographic Parameters and Reference Ranges.

Echocardiographic Parameter (Unit)	Reference Range (units)
Left Ventricular Mass Index (LVMI, g/m²)	<115 g/m² (Men), <95 g/m² (Women)
Left Ventricular Ejection Fraction (LVEF, %)	≥52% (Men), ≥54% (Women)
Left Ventricular End-Diastolic Diameter (LVEDD, mm)	42–59 mm (Men), 39–53 mm (Women)
Left Ventricular End-Systolic Diameter (LVESD, mm)	25–40 mm (Men), 22–35 mm (Women)
Left Atrial Volume Index (LAVI, mL/m²)	<34 mL/m²
Left Atrial Diameter (LAD, mm)	≤40 mm (Men), ≤38 mm (Women)
E/A Ratio (Early to Atrial transmitral flow velocities)	1.0–2.0 (Unitless, for age <60 years)
E/e′ Ratio (Transmitral E velocity to Tissue Doppler e′ velocity)	<8 (Normal, Unitless), >15 (Elevated, Unitless)
Pulmonary Artery Systolic Pressure (PASP, mmHg)	≤35 mmHg
Tricuspid Annular Plane Systolic Excursion (TAPSE, mm)	≥17 mm
Tricuspid Regurgitation Velocity (TR velocity, m/s)	<2.8 m/s
Global Longitudinal Strain (GLS, %)	< –18%

**Figure 1:**
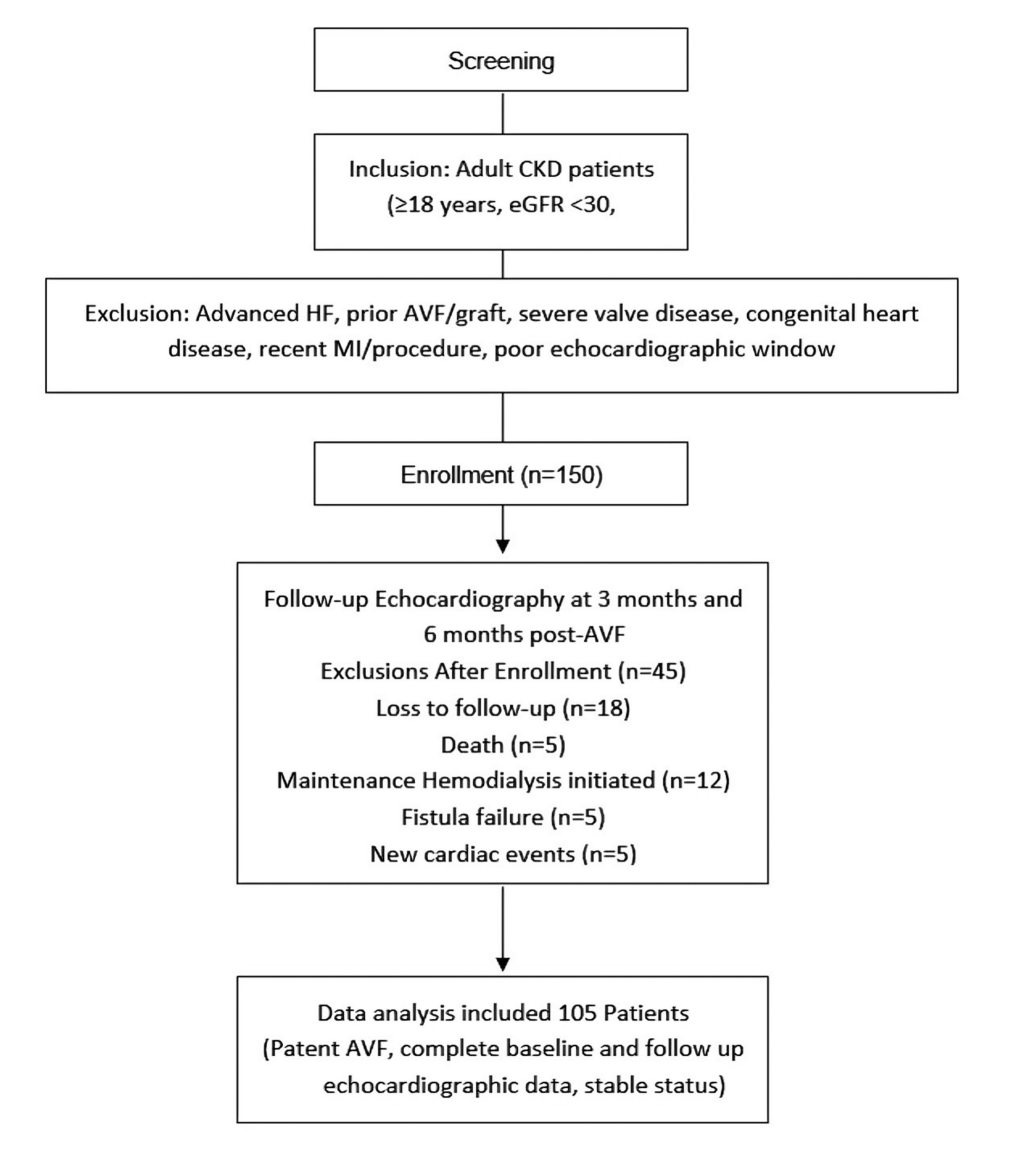
Flowchart of Patient Enrollment, Exclusions, and Final Analysis for the AVF Echocardiographic Study. CKD: Chronic kidney disease; HF: heart failure; eGFR: estimated glomerular filtration rate; AVF: arteriovenous fistula

AVF creation was carried out by a vascular surgeon after preoperative mapping, with the fistula site (radiocephalic, brachiocephalic, brachiobasilic) documented. At three and six months, follow-up echocardiography was repeated using the same protocol. Of the 150 patients initially enrolled, 45 were excluded due to loss to follow-up, death, initiation of maintenance hemodialysis, fistula failure, or new cardiac events, resulting in 105 patients with patent AVFs, complete echocardiographic data, and stable clinical status included in the final analysis. Fistula failure was defined as the inability to achieve or maintain functional patency for hemodialysis, evidenced by the absence of palpable thrill or audible bruit on clinical examination, or documented failure to mature as confirmed by vascular ultrasound. The participant selection process is summarized in Figure 1.

This was done to ensure that only patients with functioning AVFs and without confounding clinical events were included for accurate assessment of AVF-related cardiac changes. Data analysis was done using IBM SPSS Statistics for Windows, Version 26 (Released 2019; IBM Corp., Armonk, New York, United States). Comparisons between pre- and post-AVF echocardiographic parameters were made using paired t-tests. A two-sided p-value of <0.05 was considered statistically significant.

## Results

The study included 105 patients with a mean age of 42.4 ± 14.5 years. The study population consisted of 55 male patients (52.4%) and 50 female patients (47.6%). Hypertension was identified in 47 patients (44.8%), and diabetes mellitus in 36 patients (34.3%). Ischemic heart disease was found in 16 individuals (15.2%). Among types of vascular access, radiocephalic fistulas were present in 43 cases (41.0%), brachiocephalic in 44 cases (41.9%), and basilic in 18 cases (17.1%). The mean age was 42.4 years. The average eGFR was 19.9 mL/min/1.73m², consistent with advanced CKD (Table 2).

**Table 2:** Baseline Clinical and Demographic Characteristics of the Study Population (n = 105).

Variable	Frequency (%)
Gender	
Male	55 (52.4%)
Female	50 (47.6%)
Hypertension	
Yes	47 (44.8%)
No	58 (55.2%)
Diabetes Mellitus	
Yes	36 (34.3%)
No	69 (65.7%)
Ischemic Heart Disease	
Yes	16 (15.2%)
No	89 (84.8%)
AV Fistula Type	
Radiocephalic	43 (41.0%)
Brachiocephalic	44 (41.9%)
Basilic	18 (17.1%)
Continuous variables (Mean ± SD)	
Age (years)	42.4 ± 14.5
Estimated Glomerular Filtration Rate (mL/min/1.73m²)	19.9 ± 5.8

At the three-month follow-up after AVF creation, the echocardiographic assessment demonstrated clear trends in cardiac remodeling and early functional changes. An increase in the left ventricular mass index (LVMI) was observed, accompanied by modest enlargement of both left ventricular end-diastolic (LVEDD) and end-systolic diameters (LVESD), reflecting the impact of increased circulatory volume following AVF establishment. Notably, the LVEF improved, suggesting an initial adaptive augmentation in systolic function in response to elevated preload. The study population also showed an increase in the left atrial volume index (LAVI) and left atrial diameter (LAD), consistent with volume-induced atrial dilation. The early diastolic indices presented a subtle decline in the E/A ratio and a modest reduction in the E/e′ ratio, together indicating a transient enhancement in ventricular relaxation. Pulmonary artery systolic pressure (PASP) rose slightly, while tricuspid annular plane systolic excursion (TAPSE) showed a mild decrease, reflecting early right ventricular involvement. There was a small increase in tricuspid regurgitation (TR) velocity, and GLS demonstrated a minor improvement, supporting an early compensatory response. Collectively, these findings indicate that AVF creation initiates an early adaptive cardiac response characterized by increased chamber volumes, enhanced systolic performance, and mild elevations in pulmonary pressures. All comparisons showed strong correlations (r > 0.97). Detailed results are mentioned in Table 3.

**Table 3:** Comparison of Pre- and Three-Month Post-AVF Creation Echocardiographic Parameters (n = 105). SD: Standard deviation; CI: Confidence interval; r: Pearson correlation coefficient. A paired t-test was used for pre- vs post-AVF comparisons. A p-value <0.05 was considered statistically significant.

Echocardiographic Parameter (Unit)	Pre-AVF Mean ± SD	Post-AVF Mean ± SD	Mean Difference ± SD	95% CI of Difference	t-Value	p-Value	Correlation (r)
Left Ventricular Mass Index (LVMI, g/m²)	109.77 ± 12.16	120.85 ± 14.94	11.08 ± 4.01	10.31 to 11.86	28.36	<0.001	0.977
Left Ventricular Ejection Fraction (LVEF, %)	53.16 ± 5.21	56.23 ± 4.85	3.07 ± 0.72	2.93 to 3.21	43.40	<0.001	0.992
Left Ventricular End-Diastolic Diameter (LVEDD, mm)	48.43 ± 1.91	50.60 ± 2.14	2.17 ± 0.40	2.09 to 2.25	55.17	<0.001	0.987
Left Ventricular End-Systolic Diameter (LVESD, mm)	32.35 ± 2.12	33.37 ± 2.11	1.02 ± 0.14	0.99 to 1.05	76.03	<0.001	0.998
Left Atrial Volume Index (LAVI, mL/m²)	43.99 ± 5.29	46.84 ± 5.62	2.85 ± 0.62	2.73 to 2.97	47.31	<0.001	0.995
Left Atrial Diameter (LAD, mm)	35.86 ± 3.42	37.96 ± 3.27	2.10 ± 0.31	2.05 to 2.16	70.09	<0.001	0.997
E/A Ratio (Early to Atrial transmitral flow velocities)	1.06 ± 0.13	0.98 ± 0.10	–0.08 ± 0.03	–0.09 to –0.08	–31.20	<0.001	0.991
E/E' Ratio (Transmitral E velocity to Tissue Doppler e’ velocity)	13.55 ± 2.53	12.28 ± 2.33	–1.27 ± 0.29	–1.33 to –1.21	–45.02	<0.001	0.996
Pulmonary Artery Systolic Pressure (PASP, mmHg)	39.61 ± 9.85	41.87 ± 9.43	2.26 ± 0.81	2.10 to 2.41	28.59	<0.001	0.997
Tricuspid Annular Plane Systolic Excursion (TAPSE, mm)	24.19 ± 3.29	23.14 ± 3.25	–1.05 ± 0.32	–1.11 to –0.99	–33.37	<0.001	0.995
Tricuspid Regurgitation Velocity (TR velocity, m/s)	2.63 ± 0.17	2.74 ± 0.19	0.10 ± 0.13	0.08 to 0.13	8.41	<0.001	0.745
Global Longitudinal Strain (GLS, %)	–15.21 ± 0.91	–15.38 ± 0.89	–1.72 ± 0.10	–1.91 to –1.53	–18.11	<0.001	0.994

At six months post-AVF creation, significant changes were observed across all echocardiographic parameters. The LVMI increased by 20.63 ± 6.04 g/m² (p < 0.001), while the LVEF decreased by 5.32 ± 1.01% (p < 0.001). The LVEDD and LVESD increased by 4.36 ± 0.80 mm and 2.03 ± 0.17 mm, respectively (p < 0.001). The LAVI and LAD also increased significantly (p < 0.001). Diastolic dysfunction progressed, with a decrease in early-to-atrial mitral inflow velocity ratio (E/A ratio) and an increase in early mitral inflow to mitral annular velocity ratio (E/e′). PASP and TR velocity rose, while TAPSE and GLS declined (Table 4).

**Table 4:** Paired Comparison of Echocardiographic Parameters Before and Six Months After AVF Creation (n=105). Each parameter is displayed with mean and standard deviation (SD) values, mean paired difference, 95% confidence interval (CI) of the difference, t-statistic, p-value, and Pearson correlation coefficient (r). Significance threshold: p < 0.05.

Echocardiographic Parameter	Pre-AVF (Mean ± SD)	6M Post-AVF (Mean ± SD)	Mean Difference ± SD	95% CI of Difference	t-test	p-Value	Pearson r-Value
Left Ventricular Mass Index (LVMI, g/m²)	109.77 ± 12.16	130.40 ± 16.98	20.63 ± 6.04	19.46 to 21.79	34.98	<0.001	0.968
Left Ventricular Ejection Fraction (LVEF, %)	53.16 ± 5.21	47.84 ± 5.59	-5.32 ± 1.01	-5.52 to 5.13	-53.79	<0.001	0.985
Left Ventricular End-Diastolic Diameter (LVEDD, mm)	48.43 ± 1.91	52.79 ± 2.42	4.36 ± 0.80	4.21 to 4.51	56.00	<0.001	0.960
Left Ventricular End-Systolic Diameter (LVESD, mm)	32.35 ± 2.12	34.38 ± 2.11	2.03 ± 0.17	2.00 to 2.06	124.18	<0.001	0.997
Left Atrial Volume Index (LAVI, mL/m²)	43.99 ± 5.29	51.49 ± 6.41	7.50 ± 1.23	7.26 to 7.73	62.26	<0.001	0.996
Left Atrial Diameter (LAD, mm)	35.86 ± 3.42	39.44 ± 3.68	3.58 ± 0.50	3.48 to 3.68	74.01	<0.001	0.994
E/A Ratio (Early to Atrial transmitral flow velocities)	1.06 ± 0.13	0.89 ± 0.10	-0.17 ± 0.03	-0.18 to -0.17	-54.82	<0.001	0.982
E/E' Ratio (Transmitral E velocity to Tissue Doppler e’ velocity)	13.55 ± 2.53	14.42 ± 3.31	0.87 ± 0.86	0.70 to 1.04	10.36	<0.001	0.992
Pulmonary Artery Systolic Pressure (PASP, mmHg)	39.61 ± 9.85	49.50 ± 9.98	9.90 ± 1.42	9.62 to 10.17	71.38	<0.001	0.990
Tricuspid Annular Plane Systolic Excursion (TAPSE, mm)	24.19 ± 3.29	22.12 ± 3.32	-2.07 ± 0.44	-2.15 to -1.98	-47.66	<0.001	0.991
Tricuspid Regurgitation Velocity (TR velocity, m/s)	2.63 ± 0.17	3.01 ± 0.23	0.38 ± 0.14	0.35 to 0.41	28.40	<0.001	0.799
Global Longitudinal Strain (GLS, %)	-15.21 ± 0.91	-14.83 ± 0.88	0.38 ± 0.11	0.36 to 0.40	35.06	<0.001	0.993

## Discussion

The results demonstrated a progressive pattern of cardiac remodeling, with significant increases in the LVMI, LAVI, and PASP, alongside gradual declines in GLS and TAPSE. This suggests that AVF creation imposes a sustained hemodynamic burden on the cardiovascular system. A biphasic response was observed, with early improvements in LVEF, GLS, and diastolic indices, followed by significant deterioration by six months. The transition from initial compensation to maladaptive remodeling was reflected in rising LVMI, LAVI, and PASP. Notably, a proportion of patients crossed clinical thresholds for reduced LVEF and elevated PASP; however, no cases of overt heart failure were observed during follow-up. These findings underscore the importance of regular cardiac surveillance in advanced CKD after AVF creation.

A significant increase in the LVMI was observed in the present study (∆ +20.63 ± 6.04 g/m², p < 0.001), indicating progressive cardiac remodeling. This finding aligns with other studies documenting similar trends, such as that of Stoumpos et al. reporting a notable rise in LVMI from 83.7 ± 27.1 to 88.8 ± 26.2 g/m² at six weeks post-AVF (p = 0.005), with more pronounced hypertrophy in the ≥600 mL/min subgroup (↑ from 80.8 to 90.3 g/m², p < 0.001), emphasizing flow-dependent remodeling [11]. Similarly, Kim et al. observed higher baseline LVMI values in the high Qa/CO group (120.1 ± 30.8 g/m² vs. 112.1 ± 28.7 g/m²), reinforcing the role of arteriovenous flow magnitude as a determinant of hypertrophic response [13]. In contrast, studies assessing flow reduction or ligation showed reversibility. Movilli et al. noted a decrease from 135 ± 40 to 123 ± 35 g/m², and Valerianova et al. from 124.5 ± 32.7 to 115.3 ± 30.0 g/m² [14,15]. However, some studies showed stabilization with optimized volume control [16-18]. The aggregate evidence suggests that AVF-related hypertrophy is closely tied to flow burden and hemodynamic stress.

LVEF, a key indicator of systolic function, demonstrated diverse trajectories across the studies assessing cardiac remodeling following AVF creation or modification. The current study reported a biphasic response in LVEF, initially rising from 53.16 ± 5.21% to 56.23 ± 4.85% at three months, followed by a significant decline to 47.84 ± 5.59% by six months (p < 0.001). This trajectory aligns with early adaptive systolic enhancement seen in studies by Elsayed et al. (59.15% to 67.25%) [19], but contrasts with the consistent decline reported by Tayebi et al. reported a significant decrease in LVEF from 51.10% to 47.50% over 12 months (p < 0.001), suggestive of progressive myocardial dysfunction under chronic volume overload [17]. This deterioration aligns with findings from Fadel et al. (2014), where LVEF markedly dropped from 0.53 to 0.33 over 11 months, coupled with substantial ventricular dilatation and contractile impairment [20]. Other reported preserved LVEF in controlled-flow settings, suggesting that underlying cardiac reserve and AVF hemodynamics critically influence systolic adaptation [16]. Critically analyzing these patterns reveals that the impact of AVF on LVEF is both time- and flow-dependent.

There was a progressive rise in LVEDD and LVESD, with increases of 4.36 ± 1.63 mm and 2.03 ± 0.17 mm, respectively, over six months (p < 0.001). These trends are consistent with previous reports of chamber dilation post-AVF. Studies demonstrating progressive remodeling and adverse ventricular dilation reported significant increases in both LVEDD and LVESD. A study observed a marked rise in LVEDD from 51.32 ± 5.69 mm to 52.79 ± 6.48 mm (p < 0.001) at 12 months post-AVF creation, indicating volume overload and early ventricular remodeling [17]. Similarly, Kim et al. documented elevated LVEDD and LVESD values in patients with high Qa/CO ratios (LVEDD: 4.97 ± 0.61 cm; LVESD: 3.48 ± 0.56 cm), underscoring the deleterious impact of excessive AVF flow on left ventricular geometry [13]. In contrast, Basile et al. reported stable LVEDD values with no significant change, likely due to strict volume control [21]. Valerianova et al. reported a significant decrease in LVEDD from 52.0 ± 5.5 mm to 49.4 ± 6.2 mm (p = 0.002) and in LVESD from 34.8 ± 6.9 mm to 31.6 ± 8.0 mm (p = 0.0009) six weeks after AVF flow reduction, indicating rapid regression of AVF-induced structural changes [14]. Likewise, Auradkar et al. noted a statistically significant reduction in LVEDD after three months of maintenance hemodialysis, reflecting improved compliance and reduced preload burden [22]. These findings highlight the dynamic and time-sensitive nature of AVF-related cardiac remodeling.

The present study documented progressive left atrial remodeling, with LAVI increasing by 7.50 ± 3.22 mL/m² and LAD by 3.58 ± 1.89 mm over six months. Similar atrial enlargement has been reported by Elsayed et al., who observed LAD increasing from 37.2 ± 1.3 mm to 39.1 ± 1.1 mm [19]. Fadel et al. reported an extreme increase in LAVI from 43 to 85 mL/m², emphasizing the risk of maladaptive remodeling [20]. In contrast, stable LAVI and LAD values were possibly due to lower AVF flow and stricter volume control [16]. Reversal after flow reduction, as shown by Valerianova et al., further underscores the causal role of AVF-related volume burden [14].

In the current study, a biphasic alteration in diastolic function was observed following AVF creation. During the early post-operative period, the E/A ratio improved from 0.92 ± 0.16 to 1.00 ± 0.17 (p = 0.002) and E/e′ decreased from 12.48 ± 1.89 to 11.94 ± 2.04 (p = 0.012), indicating a transient improvement in left ventricular compliance. However, at six months, a reversal was evident, with the E/A ratio declining to 0.84 ± 0.19 and E/e′ rising significantly to 14.28 ± 2.34 (p < 0.001), consistent with evolving diastolic dysfunction. In comparison, Valerianova et al. (2021) demonstrated a significant reduction in E/A ratio from 1.10 ± 0.36 to 0.89 ± 0.44 (p = 0.001) after AVF flow reduction, alongside improvements in the LAVI, pulmonary artery systolic pressure (PASP), and LVEDD, suggesting reversal of AVF-induced diastolic stress [14]. Also, some studies described increased LAVI, PASP, and chamber dilation, reflecting worsening diastolic indices [12,13,17]. Fadel et al. (2014) similarly reported diastolic impairment using surrogate Doppler markers [20].

PASP is a key indicator of pulmonary hemodynamic burden and often rises in CKD patients following AVF creation. Unal et al. observed a similar rise from 35.1 ± 7.1 mmHg to 41.8 ± 8.6 mmHg [16]. Contrastingly, Manzur-Pineda et al. noted a reduction in right ventricular systolic pressure from 46 mmHg to 33 mmHg post-AVF in select patients [23]. Valerianova et al. demonstrated a PASP decline from 34.0 ± 8.4 mmHg to 29.8 ± 8.0 mmHg (p = 0.003) after AVF flow reduction, indicating reversibility [14]. Stoumpos et al. found no PASP change, possibly due to short follow-up and lower AVF flows [12].

Progressive impairment in GLS post-AVF creation has been variably reported. Valerianova et al. found no significant GLS improvement post-flow reduction (-15.4 ± 3.06% to -14.17 ± 3.43%; p = 0.22), possibly due to insufficient follow-up or minimal hemodynamic reversal [14]. Stoumpos et al. reported slight GLS improvement in low-flow groups, suggesting flow-dependence [12]. These contrasting patterns highlight GLS as a sensitive but variably responsive marker in AVF-induced remodeling.

The cardiac remodeling observed after AVF creation stems from increased venous return and reduced systemic vascular resistance, culminating in chronic volume overload. While early improvements in LVEF and GLS may reflect adaptive responses, prolonged exposure often leads to diastolic dysfunction, myocardial fibrosis, and chamber dilation. Thresholds such as AVF flow >600 mL/min or Qa/CO ratio >0.3 are consistently associated with worsening cardiac indices [12,14]. This study’s strengths include its prospective design, focused patient selection, and serial echocardiographic assessment, which allowed for a clear evaluation of cardiac changes following AVF creation. However, certain limitations must be acknowledged, including the lack of a control group, absence of direct AVF flow measurements, limited adjustment for confounding factors, and a modest sample size. In addition, all echocardiographic measurements were performed by a single, unblinded cardiologist, which may introduce some measurement bias. These findings support the need for routine cardiac surveillance and timely consideration of AVF flow adjustment as part of dialysis care in patients with advanced CKD, aiming to prevent adverse cardiac outcomes.

## Conclusions

This study found that creating an AVF in advanced CKD patients led to early improvement in cardiac function at three months but was followed by clear worsening in both heart structure and performance by six months. Some of these changes may be reversible with adjustments in fistula flow or medical treatment. It is recommended that patients undergo echocardiographic evaluation at baseline, three months, and six months after fistula creation, particularly if there is underlying heart disease. These findings highlight the importance of careful AVF planning and patient selection. Further, larger and controlled studies are needed to confirm these results and guide monitoring practices.
